# Prevention of Doxorubicin-Induced Autophagy Attenuates Oxidative Stress and Skeletal Muscle Dysfunction

**DOI:** 10.3390/antiox9030263

**Published:** 2020-03-23

**Authors:** Vivian Doerr, Ryan N. Montalvo, Oh Sung Kwon, Erin E. Talbert, Brian A. Hain, Fraser E. Houston, Ashley J. Smuder

**Affiliations:** 1 Department of Applied Physiology and Kinesiology, University of Florida, Gainesville, FL 32611, USA; vdoerr@ufl.edu (V.D.); ryan.montalvo@ufl.edu (R.N.M.); 2 Department of Kinesiology, University of Connecticut, Storrs, CT 06269, USA; ohsung.kwon@uconn.edu; 3 Department of Health and Human Physiology, University of Iowa, Iowa City, IA 52242, USA; erin-talbert@uiowa.edu; 4 Department of Cellular and Molecular Physiology, College of Medicine, Pennsylvania State University, Hershey, PA 17033, USA; bhain@pennstatehealth.psu.edu; 5 Department of Health Sciences and Human Performance, University of Tampa, Tampa, FL 33606, USA; fhouston@ut.edu

**Keywords:** adriamycin, antioxidant, mitochondria, peroxisome proliferator-activated receptor gamma co-activator 1-alpha, chemotherapy

## Abstract

Clinical use of the chemotherapeutic doxorubicin (DOX) promotes skeletal muscle atrophy and weakness, adversely affecting patient mobility and strength. Although the mechanisms responsible for DOX-induced skeletal muscle dysfunction remain unclear, studies implicate the significant production of reactive oxygen species (ROS) in this pathology. Supraphysiological ROS levels can enhance protein degradation via autophagy, and it is established that DOX upregulates autophagic signaling in skeletal muscle. To determine the precise contribution of accelerated autophagy to DOX-induced skeletal muscle dysfunction, we inhibited autophagy in the soleus via transduction of a dominant negative mutation of the autophagy related 5 (ATG5) protein. Targeted inhibition of autophagy prevented soleus muscle atrophy and contractile dysfunction acutely following DOX administration, which was associated with a reduction in mitochondrial ROS and maintenance of mitochondrial respiratory capacity. These beneficial modifications were potentially the result of enhanced transcription of antioxidant response element-related genes and increased antioxidant capacity. Specifically, our results showed significant upregulation of peroxisome proliferator-activated receptor gamma co-activator 1-alpha, nuclear respiratory factor-1, nuclear factor erythroid-2-related factor-2, nicotinamide-adenine dinucleotide phosphate quinone dehydrogenase-1, and catalase in the soleus with DOX treatment when autophagy was inhibited. These findings establish a significant role of autophagy in the development of oxidative stress and skeletal muscle weakness following DOX administration.

## 1. Introduction

Doxorubicin (DOX) is a highly effective chemotherapy agent, widely used in the treatment of a variety of cancers [1,2,3]. However, DOX treatment contributes to the development of skeletal muscle weakness and fatigue in cancer patients, which negatively impacts quality of life [4,5,6]. Although the mechanisms responsible for DOX-induced skeletal muscle dysfunction are unknown, reports suggest that increased reactive oxygen species (ROS) formation within the muscle plays a primary role [4,7,8,9]. Specifically, DOX accumulates within the mitochondria of skeletal muscle as a result of an affinity for the phospholipid cardiolipin [10]. Localization to the inner mitochondrial membrane elicits the reduction of DOX by nicotinamide adenine dinucleotide (NADH)-dehydrogenase of complex I, resulting in the formation of superoxide radicals that in turn enhance ROS production and mitochondrial dysfunction [8]. This increase in DOX-induced mitochondrial ROS production is associated with enhanced cytosolic and myofibrillar protein degradation in skeletal muscle, causing disruption to muscle contraction and fiber atrophy [10]. Specifically, oxidative damage to skeletal muscle acts as an upstream trigger to stimulate muscle breakdown through activation of key proteolytic systems [7,9,11,12].

In this regard, autophagy is a catabolic process that is required for the elimination of damaged cytosolic proteins and organelles [13]. Although basal levels of autophagy are required for normal muscle function, under pathological conditions autophagy can elicit a deleterious rate of protein turnover, resulting in muscular weakness [7]. Indeed, following DOX administration, it is established that autophagy is upregulated in striated muscle [14]. Although the effects of accelerated autophagy have not been fully elucidated in skeletal muscle, DOX-induced autophagy has been shown to promote cellular dysfunction and apoptosis in the heart, resulting in left ventricular dysfunction [15]. Importantly, inhibition of autophagy by both pharmacological and gene silencing interventions results in improvements in cardiac function and reductions in mitochondrial dysfunction [16,17,18,19,20].

Although overwhelming evidence supports the role of ROS in activating autophagic protein breakdown in skeletal muscle, previous work by our laboratory and others has revealed that prevention of the pathological upregulation of autophagy is sufficient to reduce cellular ROS levels in several tissue types [7,21,22]. Specifically, prevention of accelerated autophagy stimulated by prolonged diaphragm muscle inactivity mitigated mitochondrial ROS emission via augmentation of antioxidant capacity [7]. Additionally, knockdown of autophagy protein 5 (ATG5) was shown to reduce chemotherapy-induced oxidative stress in osteosarcoma cells [22]. Despite these reports, the precise effects of accelerated autophagy on skeletal muscle following DOX administration remain unknown. Therefore, we investigated whether DOX-induced enhancement of autophagic signaling in the soleus muscle is required for muscle atrophy and contractile dysfunction, and whether increased autophagy signaling plays a role in promoting skeletal muscle redox disturbances. 

## 2. Materials and Methods 

### 2.1. Animals

Adult (~4–6-month-old) female Sprague-Dawley rats were used in these experiments. Female animals were utilized because DOX is an integral component of the standard of care for breast cancer patients and is often given as single agent therapy in this patient population [23,24]. Animals were housed at the University of Florida Animal Care Services Center and maintained under a 12:12 h light/dark cycle. Food and water were provided ad libitum throughout the experimental period. The Institutional Animal Care and Use Committee of the University of Florida approved these experiments (protocol no. 201507739).

### 2.2. Experimental Design

#### 2.2.1. Experiment 1

This experiment was performed to determine the effect of dominant negative ATG5 recombinant adeno-associated virus (rAAV-dnATG5) administration on ATG12–ATG5 conjugation, soleus muscle fiber cross-sectional area (CSA), specific force production, and mitochondrial function in control animals. Animals were randomly assigned to one of two experimental groups (*n* = 8/group): (1) control animals treated with saline (Saline) and (2) control animals treated with rAAV-dnATG5 (dnATG5). Animals received an intramuscular (i.m.) injection of rAAV-dnATG5 (1 × 10^11^ vg) or saline (equal volume to rAAV-dnATG5 animals) directly into each soleus muscle [25]. The dnATG5 rAAV was injected into the soleus muscle to localize the effect of the rAAV, which was done in an effort to reduce the potential off-target effects of whole-body autophagy downregulation. Animals were sacrificed 4 weeks after soleus saline or dnATG5 treatment, and soleus muscles were removed and used for analyses. One whole soleus was immediately used to assess in vitro muscle-specific force production. From the second soleus, a portion of the muscle was prepared for histological analysis and another portion was used to assess mitochondrial respiration and ROS emission. The remaining muscle was frozen immediately in liquid nitrogen and stored at –80 °C for subsequent analysis of ATG12-ATG5 conjugation.

#### 2.2.2. Experiment 2

To test the hypothesis that prevention of DOX-induced autophagy will improve soleus muscle function, animals were randomly assigned to the following three groups: (*n* = 8/group): (1) control animals treated with saline (Saline-Saline), (2) control animals treated with DOX (Saline-DOX), and (3) rAAV-dnATG5 animals treated with DOX (dnATG5-DOX). Animals received saline or rAAV-dnATG5, as described in experiment 1. Four weeks after rAAV-dnATG5 or saline administration into each soleus muscle (i.m.), the animals received either DOX (20 mg/kg i.p.) or saline treatment (equal volume). This dose of DOX has been scaled for use in rats and is a regularly used animal dose that is within the range used for humans [26,27]. This dose is well-established to induce skeletal muscle dysfunction in animals [8,28,29]. Then, 48 h following assigned treatments, animals were sacrificed, and soleus muscles were prepared as described above for subsequent analysis.

### 2.3. Experimental Protocol

#### 2.3.1. Packaging and Purification of Recombinant AAV Vectors

The dnATG5 plasmid was purchased from Addgene (plasmid #13096) and deposited by Roberta Gottlieb. ATG12 to ATG5 conjugation is a required step in autophagosome elongation [30]. The dnATG5 plasmid has a one-base mutation that prevents the conjugation of ATG5 to ATG12. This step is necessary for light chain 3 (LC3) incorporation into the early autophagosomal structure, and thus autophagy is inhibited at the level of autophagosome formation. The pTRUF12-dnATG5 plasmid was constructed as described previously [7]. The rAAV-dnATG5 vector was generated, purified, and tittered by the University of Florida Powell Gene Therapy Center Vector Core Lab.

#### 2.3.2. Muscle Cross-Sectional Area 

Sections from frozen soleus samples were cut at 10 microns using a cryotome (Shandon Inc., Pittsburgh, PA, USA) and stained for dystrophin (Fisher Scientific, Hampton, NH, USA) myosin heavy chain (MHC) type I and MHC type IIa (Developmental Studies Hybridoma Bank, Iowa City, IA, USA) for fiber CSA analysis, as described previously [7]. CSA was determined using Scion software (version 4.0, NIH, Bethesda, MD, USA).

#### 2.3.3. In Vitro Muscle Contractile Properties 

One whole soleus was dissected and suspended vertically within a jacketed tissue bath containing Krebs–Henseleit buffer (Sigma-Aldrich, St. Louis, MO, USA) equilibrated with 95% O_2_–5% CO_2_. One end of the muscle was tied to a Dual-Mode Muscle Lever System (305C-LR, Aurora Scientific Inc., Aurora, Canada) and the other secured onto a plastic rod using 4.0 gauge nylon suture. The soleus was stimulated along its length via platinum wire electrodes using supramaximal (≈150%) stimulation voltage. The optimum contractile length (L_o_) was first determined [7]. To measure the force frequency response, the soleus was stimulated supramaximally with 120 V pulses at 15–160 Hz. Soleus-specific force production was normalized to the muscle wet weight and length, as previously described [31].

#### 2.3.4. Electron Microscopy 

Soleus muscle samples were fixed in phosphate-buffered saline (PBS) with 4% paraformaldehyde. The University of Florida Interdisciplinary Center for Biotechnology Research Electron Microscopy Core Lab treated and prepared the soleus muscle samples for imaging. Images of the soleus muscle were acquired with a Hitachi H-7000 TEM (Hitachi High-Tech, Tokyo, Japan).

#### 2.3.5. Muscle Fiber Permeabilization 

Small pieces of soleus muscle (~5–7 mg) were teased apart to separate fibers in ice-cold Buffer X (60 mM 4-Morpholineethanesulfonic acid potassium salt (K-MES), 35 mM KCl, 7.23 mM K_2_-(ethylene glycol-bis(β-aminoethyl ether)-N,N,N’,N’-tetraacetic acid) EGTA, 2.77 mM CaK_2_EGTA, 20 mM imidazole, 0.5 mM dithiothreitol (DTT), 20 mM taurine, 5.7 mM ATP, 15 mM phosphocreatine, and 6.56 mM MgCl_2_, pH 7.1) [8]. Fiber bundles were permeabilized by rotating for 30 min at 4 °C in Buffer X containing 50 μg/mL saponin. Fiber bundles were then washed three times for 5 minutes each in separate tubes containing Buffer Z (110 mM K-MES, 35 mM KCl, 1 mM EGTA, 5 mM K_2_HPO_4_, 3 mM MgCl_2_, 0.05 mM glutamate, 0.02 mM malate, and 0.5 mg/ml bovine serum albumin (BSA), pH 7.1).

#### 2.3.6. Mitochondrial Respiration 

Respiration was measured with an oxygraph (Hansatech Instruments, King’s Lynn, United Kingdom), using previously described techniques [8]. Specifically, permeabilized soleus fiber bundles were placed in respiration chambers maintained at 37 °C, with 1 mL of Buffer Z containing 20 mM creatine to saturate creatine kinase. A total of 5 mM of pyruvate and 5 mM of malate were added to measure flux through complex I. State 3 was initiated by adding 0.25 mM ADP to the chamber. State 4 was determined by adding 10 μg/mL oligomycin to inhibit ATP synthesis. The respiratory control ratio (RCR) was calculated by dividing state 3 by state 4 respiration.

#### 2.3.7. Mitochondrial ROS Emission 

ROS emission in permeabilized soleus muscle fibers was determined using Amplex Red (Molecular Probes, Eugene, OR, USA). Details of this assay have been described previously [8]. Sample fluorescence was measured after 30 minutes of incubation at 37 °C. The fluorescence was normalized to dry weight of the tissue to control for the size of the muscle fiber bundle.

#### 2.3.8. DOX Concentration 

The relative concentration of DOX in the soleus muscle was assessed using a commercially available Rat Adriamycin ELISA kit according to the manufacturer’s instructions (MyBioSource, San Diego, CA, USA). 

#### 2.3.9. Western Blot Analysis 

Soleus muscle was homogenized and assayed as described [32]. Briefly, soleus tissues were homogenized 1:10 (*w/v*) in buffer containing 5 mM Tris (pH 7.5) and 5 mM ethylenediaminetetraacetic acid (EDTA) (pH 8.0) with a protease inhibitor cocktail (Sigma-Aldrich, St. Louis, MO, USA). Homogenate was centrifuged at 1500× *g* for 10 min at 4 °C. The pellet and supernatant were separated. The Bradford method (Sigma-Aldrich, St. Louis, MO, USA) was used to assess protein content. Proteins were separated via 4–20% precast gels (Bio-Rad Laboratories, Hercules, CA, USA) and transferred to nitrocellulose membranes, which were probed with primary antibodies for the proteins of interest: ATG12-ATG5 #4180, LC3B #2775 phosphorylated-eukaryotic translation initiation factor 2 subunit 1 (peIF2α)-S51 #9721, total-eIF2α #9722, activating transcription factor 4 (ATF4) #11815, C/EBP homologous protein (CHOP) #2895 (Cell Signaling Technologies, Danvers, MA, USA), glutathione peroxidase 1 (GPX1) #ab22604, catalase #ab16731, citrate synthase #ab96600, peroxisome membrane protein 70 (PMP70) #ab3421 (Abcam, Cambridge, United Kingdom), superoxide dismutase 1 (SOD1) #sc11407, and superoxide dismutase 2 (SOD2) #sc30080 (Santa Cruz Biotechnology, Dallas, TX, USA). Protein abundances were normalized to glyceraldehyde 3-phosphate dehydrogenase (GAPDH) #sc47724 (Santa Cruz Biotechnology, Dallas, TX, USA), which served as a loading control. Following overnight incubation with primary antibodies at 4 °C, membranes were washed extensively with phosphate-buffered saline with 0.05% Tween and then incubated with corresponding secondary antibodies (anti-mouse IgG #7076S, anti-rabbit IgG #7074S; Cell Signaling Technologies, Danvers, MA, USA). Membranes were imaged using enhanced chemiluminescence 2 (ECL2) Western Blotting Substrate (Thermo Fisher Scientific, Waltham, MA, USA) and captured using the G:Box imager (Syngene, Frederick, MD, USA). Images were then analyzed using ImageJ software (version 1.51, NIH, Bethesda, MD, USA).

#### 2.3.10. RNA Isolation and cDNA Synthesis 

Total RNA was isolated from soleus muscle tissue with TRIzol Reagent (Thermo Fisher Scientific, Waltham, MA, USA) according to the manufacturer’s instructions. Total RNA and RNA content (µg/mg muscle) were evaluated by spectrophotometry. Total RNA (5 µg) was then reverse transcribed with the Superscript III First-Strand Synthesis System for RT-PCR (Thermo Fisher Scientific, Waltham, MA, USA), using oligo(dT)20 primers and the protocol outlined by the manufacturer.

#### 2.3.11. Real-Time Polymerase Chain Reaction 

One microliter of cDNA was added to a 24 μL PCR reaction for real-time PCR using Taqman chemistry, and quantification was determined using the Applied Biosystems 7300 Real Time PCR System and software (ABI, Foster City, CA, USA). mRNA expression of x-box-binding protein 1 (*XBP1), CHOP, GPX1, SOD1, SOD2, catalase,* sirtuin 1 *(SIRT1),* sirtuin 3 *(SIRT3),* proliferator-activated receptor gamma co-activator 1-alpha (*PGC-1α),* nicotinamide-adenine dinucleotide phosphate (NAD(P)H) quinone dehydrogenase-1 (*NQO1*)*,* 8-oxoguanine DNA glycosylase (*OGG-1*)*,* heme oxygenase-1 (*HO-1*)*,* nuclear respiratory factor-1 (*NRF1*)*,* and nuclear factor erythroid-2-related factor-2 (*NRF2*) were analyzed. mRNA transcripts were assayed using predesigned rat primer and probe sequences (Thermo Fisher Scientific, Waltham, MA, USA). On the basis of previous work showing unchanged expression with experimental manipulations, β-glucuronidase (*GusB*) was used as the housekeeping gene for soleus muscle samples [7].

### 2.4. Statistical Analysis

A Student’s *t*-test was used to compare groups in experiment 1. One-way analysis of variance (ANOVA) was used to make comparisons between groups for each dependent variable in experiment 2, and a Tukey’s HSD (honestly significant difference) test was performed post-hoc. Significance was established at *p* < 0.05. Data are presented as means ± standard error of mean (SEM).

## 3. Results

### 3.1. Experiment 1

#### Physiological Response to rAAV-dnATG5 Treatment

**Figure 1:**
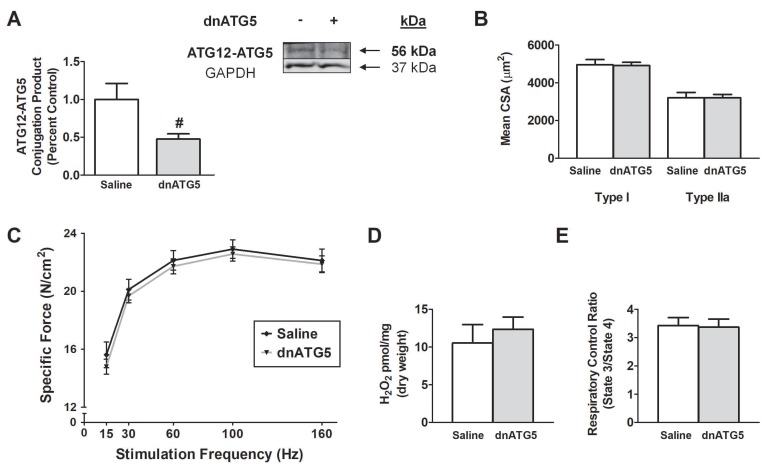
Effect of dominant negative ATG5 recombinant adeno-associated virus (rAAV-dnATG5) administration on: (**A**) ATG12-ATG5 conjugation, (**B**) soleus muscle fiber cross-sectional area (CSA), (**C**) soleus muscle-specific force production, (**D**) mitochondrial hydrogen peroxide (H_2_O_2_) emission, and (**E**) mitochondrial respiratory control ratio (RCR). Values are represented as means ± SEM. ^#^ significantly different versus Saline (*p* < 0.05).

To determine the impact of rAAV-dnATG5 administration on the soleus muscle, animals received an intramuscular injection of saline or rAAV-dnATG5 4 weeks prior to muscle analysis. Our results demonstrated that, compared to Saline animals, the dnATG5 treatment significantly reduced basal ATG12-ATG5 conjugation (Figure 1A). The decrease in basal ATG12-ATG5 levels did not impact soleus muscle fiber CSA or specific force production as no differences existed between Saline and dnATG5 animals (Figure 1B,C). Importantly, dnATG5 also had no effect on basal ROS production or mitochondrial function compared to Saline (Figure 1D,E). As no adverse effects were caused by rAAV-dnATG5 administration into the soleus muscle, this group was not included in further analyses. This led to the second experiment, to determine if reducing DOX-induced autophagy to basal levels would prevent soleus muscle dysfunction.

### 3.2. Experiment 2

#### 3.2.1. Systemic Effects of DOX on Body Weight and Soleus Mass

No difference in body weight existed between animals prior to Saline or rAAV-dnATG5 treatment (initial), prior to Saline or DOX administration (treatment) or 2 days following treatment (final) (Table 1). In addition, absolute and relative soleus muscle weight was maintained between all groups.

#### 3.2.2. rAAV-dnATG5 Prevented ATG12-ATG5 Conjugation and Reduced Autophagosome Formation in DOX-Treated Animals

**Figure 2:**
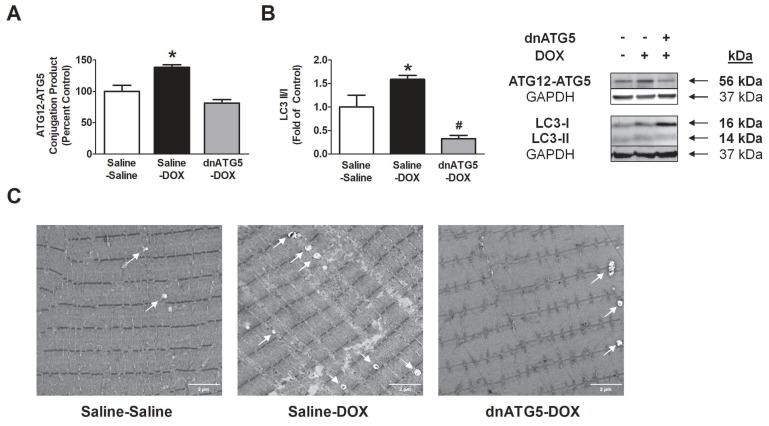
Protein expression of (**A**) the ATG12-ATG5 conjugation product and (**B**) light chain 3 (LC3)-II/I ratio. Values are represented as means ± SEM. Representative Western blot images are shown to the right of the graph. * significantly different versus all groups (*p* < 0.05). ^#^ significantly different versus Saline-Saline (*p* < 0.05). (**C**) Representative soleus muscle electron microscopy images. White arrows within each soleus muscle image indicate autophagic vacuoles. Soleus ultrastructure appears identical between the Saline-Saline and dnATG5-doxorubicin (DOX) groups.

Protein expression of the ATG12-ATG5 conjugation product was assessed in each experimental group to confirm the effectiveness of our rAAV-dnATG5 dosing protocol. Our results demonstrated that DOX administration significantly increased ATG12-ATG5 conjugation in the soleus, and that administration of rAAV-dnATG5 with DOX successfully prevented this increase (Figure 2A). Importantly, no differences existed in expression of the ATG12-ATG5 conjugation product between the Saline-Saline and dnATG5-DOX groups. Furthermore, we saw an increase in autophagic signaling with DOX treatment, as shown by elevation of the LC3-II/I ratio compared to both the Saline-Saline and dnATG-DOX groups (Figure 2B). Additionally, electron microscopy was used to visualize autophagosome formation in the soleus muscle. These representative images (Figure 2C) illustrate a DOX-dependent increase in autophagic vacuole formation that is attenuated in dnATG5-DOX animals. Vacuole formation appeared to be reduced in dnATG5-DOX animals compared to Saline-Saline animals.

#### 3.2.3. Autophagy Inhibition Prevented DOX-Induced Soleus Muscle Weakness and Mitochondrial Dysfunction

**Figure 3:**
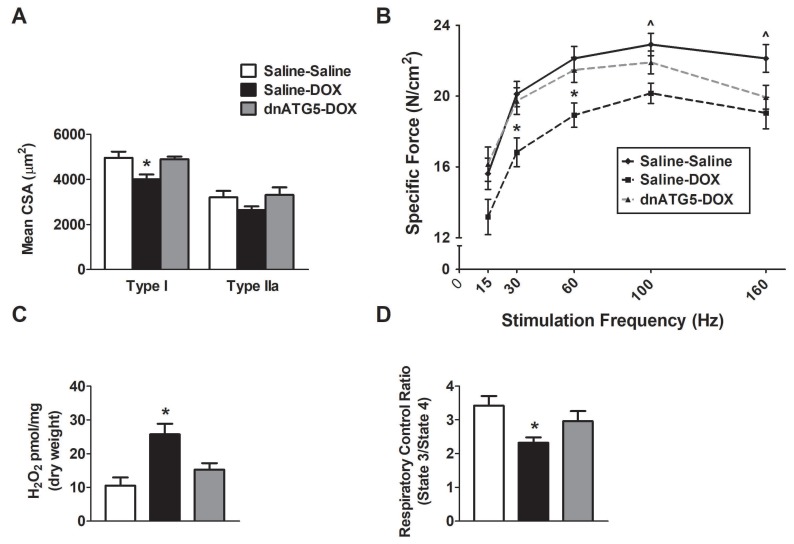
Effects of DOX and rAAV-dnATG5 administration on (**A**) soleus muscle fiber cross-sectional area (CSA), (**B**) soleus muscle-specific force production, (**C**) mitochondrial hydrogen peroxide (H_2_O_2_) emission, and (**D**) mitochondrial respiratory control ratio (RCR). Values are represented as means ± SEM. * significantly different versus all groups (*p* < 0.05). ^ significantly different versus Saline-DOX (*p* < 0.05).

DOX administration in the saline-treated animals resulted in a significant reduction in the CSA of type I soleus muscle fibers compared to both the Saline-Saline and dnATG5-DOX groups (Figure 3A). rAAV-dnATG5 transduction in the soleus prior to DOX treatment prevented soleus muscle atrophy of type I fibers. No difference in type IIa fibers existed between groups. In addition, DOX treatment also altered the soleus muscle force frequency response (Figure 3B). Specific force production of the soleus muscle was impaired at stimulation frequencies of 30–160 Hz in Saline-DOX animals compared to Saline-Saline animals. At stimulation frequencies of 100 and 160 Hz, soleus muscle force production of the dnATG5-DOX animals was not significantly different compared to either the Saline-Saline or the Saline-DOX group. However, muscle contractile force was preserved in dnATG5-DOX animals compared to Saline-DOX at stimulation frequencies of 30 and 60 Hz.

Assessment of mitochondrial ROS production from permeabilized soleus muscle fiber bundles revealed a significant increase in hydrogen peroxide (H_2_O_2_) emission from Saline-DOX animals compared to Saline-Saline (Figure 3C). Additionally, our results showed a significant reduction in the mitochondrial respiratory control ratio in Saline-DOX animals compared to Saline-Saline (Figure 3D). This DOX-induced increase in ROS emission and reduction in soleus mitochondrial efficiency was attenuated in the dnATG5-DOX animals. 

#### 3.2.4. DOX did not Enhance Endoplasmic Reticulum Stress Signaling in the Soleus

**Figure 4:**
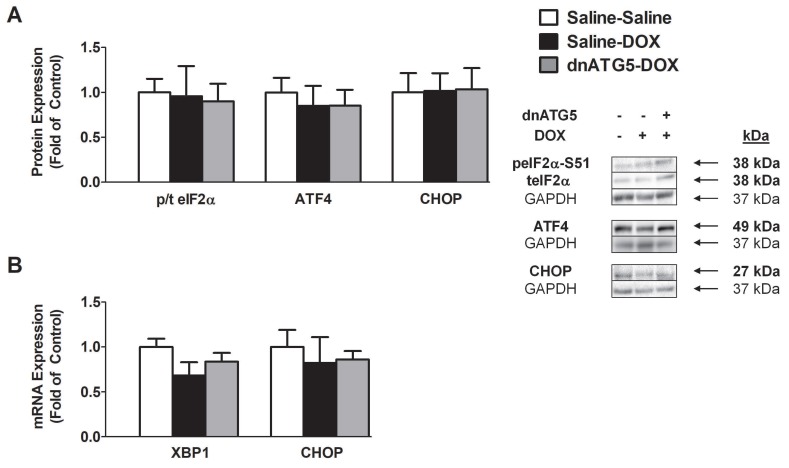
Markers of endoplasmic reticulum stress in the soleus muscle. (**A**) Ratio of phosphorylated to total eukaryotic translation initiation factor 2 subunit 1 (eIF2α) (p/t eIF2α), activating transcription factor 4 (ATF4) protein expression, and C/EBP homologous protein (CHOP) protein expression. (**B**) mRNA expression of x-box binding protein 1 (XBP1) and CHOP. Values are represented as means ± SEM. Representative Western blot images are shown to the right of the graph.

The endoplasmic reticulum (ER) stress response can modulate autophagy via increased eIF2α phosphorylation, which stimulates the upregulation of autophagy genes [33]. However, our results demonstrated that enhanced autophagy signaling following DOX administration does not occur as a result of activation of the canonical PERK/eIF2α-ATF4-CHOP pathway. Specifically, no differences existed in the phosphorylation of eIF2α, the protein expression of ATF4 and CHOP, and the mRNA expression of XBP1 and CHOP between any of the treatment groups (Figure 4A,B). 

#### 3.2.5. Citrate Synthase Protein Expression was Increased when Autophagy was Inhibited in DOX-Treated Animals 

**Figure 5:**
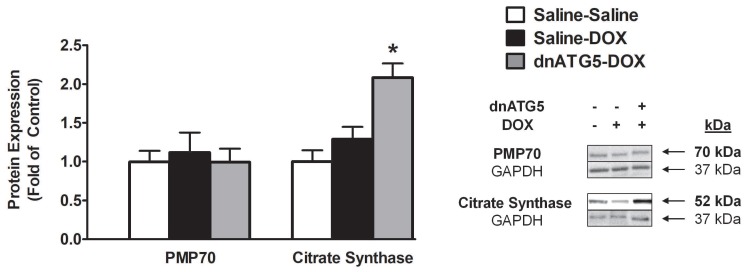
Protein expression of peroxisome membrane protein 70 (PMP70) and citrate synthase. Representative Western blot images are shown to the right of the graph. Values are represented as means ± SEM. * significantly different versus all groups (*p* < 0.05).

Skeletal muscle dysfunction prompted by accelerated autophagy may potentially be linked to altered rates of peroxisome and mitochondria removal [7]. To determine if DOX-induced autophagy modifies the concentration of these organelles within the soleus muscle, PMP70 and citrate synthase were assessed. Our results demonstrated that DOX treatment does not result in enhanced peroxisome degradation as no differences existed in the protein expression of PMP70 between groups. However, citrate synthase expression was significantly increased in the dnATG5-DOX animals compared to all other groups (Figure 5).

#### 3.2.6. DOX Accumulation within the Soleus Muscle Was not Affected by Autophagy Inhibition

**Figure 6:**
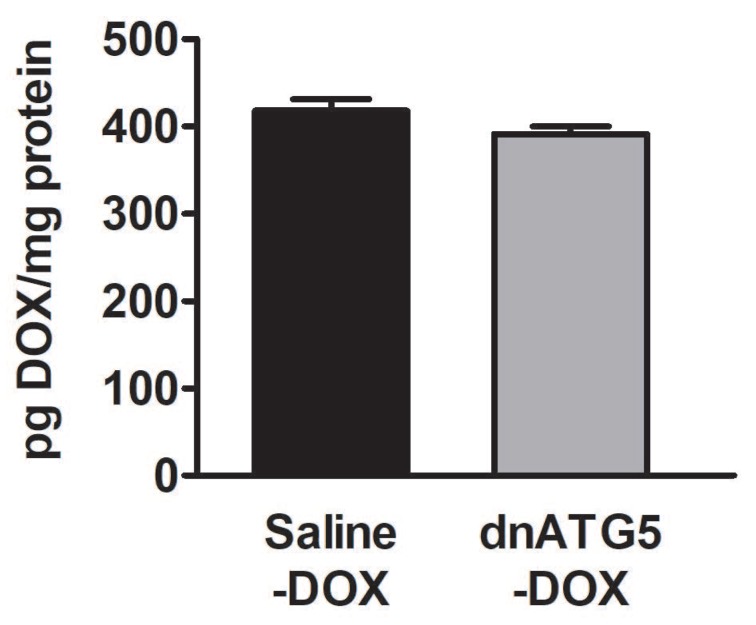
The relative concentration of DOX in the soleus muscle. Values are represented as means ± SEM.

It is established that DOX preferentially localizes within the mitochondria and that muscle tissue with greater mitochondrial volume appear to accumulate greater levels of DOX [34]. In addition, levels of DOX accumulated within the muscle tissue may be associated with the degree of skeletal muscle dysfunction [10]. Therefore, we also assessed the concentration of DOX within the soleus muscle to determine if changes in citrate synthase corresponded to differences in the level of DOX accumulated within the soleus of the Saline-DOX and dnATG5-DOX groups. Our data showed no differences in DOX accumulation in whole soleus homogenate from DOX animals treated with saline or rAAV-dnATG5 (Figure 6).

#### 3.2.7. Prevention of DOX-Induced Autophagy Enhanced Soleus Muscle Antioxidant Capacity

**Figure 7:**
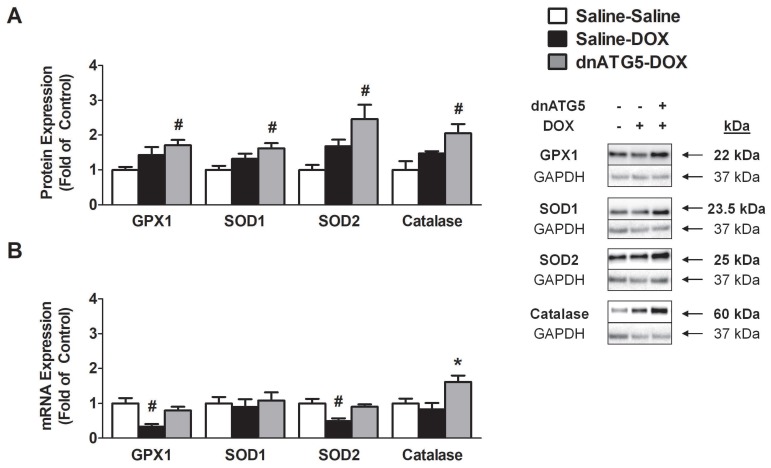
Endogenous antioxidant enzyme expression in the soleus. (**A**) Protein expression of glutathione peroxidase 1 (GPX1), superoxide dismutase 1 (SOD1), superoxide dismutase 2 (SOD2), and catalase. (**B**) mRNA expression of GPX1, SOD1, SOD2, and catalase. Values are represented as means ± SEM. Representative Western blot images are shown to the left of the graph. * significantly different versus all groups (*p* < 0.05). # significantly different versus Saline-Saline (*p* < 0.05).

Soleus muscle antioxidant capacity was measured via assessment of the protein and mRNA expression of the endogenous antioxidant enzymes GPX1, SOD1, SOD2, and catalase. Western blot analysis revealed a significant increase in the soleus expression of each protein in the dnATG5-DOX group compared to the Saline-Saline animals (Figure 7A). Gene expression of each antioxidant differed from protein expression as GPX1 and SOD2 expression was significantly reduced in the Saline-DOX animals compared to Saline-Saline, no differences existed between groups for SOD1, and furthermore catalase gene expression was increased in dnATG5-DOX animals compared to all groups (Figure 7B). 

#### 3.2.8. Inhibition of DOX-Induced Autophagy Upregulated Proteins Responsible for Mitochondrial Quality Control and Adaptation to Oxidative Stress

**Figure 8:**
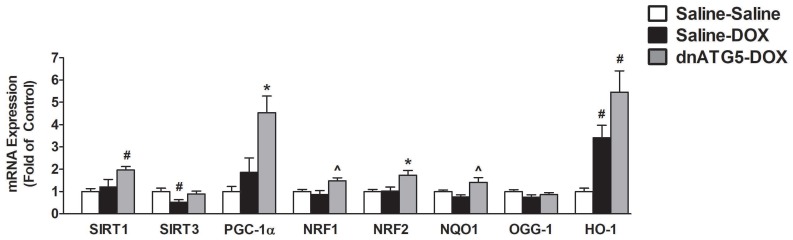
Markers of mitochondrial volume and the antioxidant response element signaling. mRNA expression of sirtuin 1 (SIRT1), sirtuin 3 (SIRT3), peroxisome proliferator-activated receptor gamma co-activator 1-alpha (PGC-1α), nuclear respiratory factor-1 (NRF1), nuclear factor erythroid-2-related factor-2 (NRF2), NAD(P)H quinone dehydrogenase-1 (NQO1), 8-oxoguanine DNA glycosylase (OGG-1), and heme oxygenase-1 (HO-1). Values are represented as means ± SEM. * significantly different versus all groups (*p* < 0.05). # significantly different versus Saline-Saline (*p* < 0.05). ^ significantly different versus Saline-DOX (*p* < 0.05).

The histone deacetylases SIRT1 and SIRT3 have been implicated in the regulation of mitochondrial turnover and the antioxidant response via activation of PGC-1α, NRF1 and NRF2 signaling [35]. In this regard, our results showed a significant increase in SIRT1 in dnATG5-DOX animals compared to Saline-Saline, and a reduction in SIRT3 levels in Saline-DOX animals compared to Saline-Saline (Figure 8). In addition, the expression of PGC-1α and NRF2 was elevated in the dnATG5-DOX group compared to all others, whereas NRF1 was also increased in the dnATG5-DOX group compared to Saline-Saline. NRF2 can lead to enhanced antioxidant defense through activation of the antioxidant response element (ARE) signaling pathway [36], and our results showed that NQO1 was elevated in the dnATG5-DOX group compared to Saline-DOX. In addition, HO-1 was upregulated in both the dnATG5-DOX and Saline-DOX groups compared to Saline-Saline. Finally, no changes were seen in the mRNA expression of OGG-1 between treatment groups.

## 4. Discussion

### 4.1. Inhibition of Autophagy Prevented DOX-Induced Skeletal Muscle Atrophy and Dysfunction

Decreases in skeletal muscle function and strength are a hallmark of cancer patients following chemotherapy treatment, and it is established that loss of muscle mass negatively affects patient outcomes [37,38]. Preclinical studies investigating the skeletal muscle response to DOX therapy parallel these findings and demonstrate that, independent of cancer burden, DOX administration promotes muscle atrophy and weakness [4,8,39]. Our results confirm these previous findings and demonstrate a significant reduction in soleus muscle CSA of type I fibers and in soleus muscle-specific force production following DOX exposure. Specifically, a recent report by our group demonstrated a similar reduction in soleus muscle fiber CSA and contractile function [29], and assessment of the force-frequency response in the soleus muscle following DOX exposure by Ertunc et al. also revealed significant contractile dysfunction at both submaximal and maximal stimulation frequencies [40]. Evidence from these reports suggest that these effects may be the result of disruption to signal transmission at the neuromuscular junction and/or intracellular calcium release and uptake [40]. However, the signaling pathways responsible for DOX-induced soleus muscle dysfunction remain unclear. Our laboratory and others have reported the significant upregulation of several proteins required for elevated protein breakdown via autophagy acutely following DOX administration [12,14,41,42,43]. These initial findings suggest a role for enhanced autophagy signaling in the development of DOX-induced skeletal muscle weakness, and our results confirm that the upregulation of autophagy by DOX contributes to increased muscle proteolysis and contractile dysfunction. Specifically, inhibition of pathological autophagy activation in the soleus muscle prevented muscle fiber atrophy and the reduction in specific force production at submaximal frequencies. Indeed, this study provides the first piece of evidence directly linking autophagy to DOX-induced skeletal muscle weakness. 

### 4.2. DOX-Induced Autophagy Promoted Soleus Muscle Dysfunction and ROS Production Independent of Changes in Endoplasmic Reticulum Stress

Oxidative damage to skeletal muscle proteins as a result of increased mitochondrial ROS production has been demonstrated to increase autophagy through the disruption of ER homeostasis and activation of the unfolded protein response (UPR) [44]. With regard to muscle wasting, activation of the UPR has been shown to contribute to cancer cachexia-induced skeletal muscle atrophy, primarily through the induction of eIF2α phosphorylation and splicing of XBP1 [45]. Activation of eIF2α induces the expression of the transcription factor ATF4, which leads to the activation of several genes involved in the adaptation to cellular stress [12,33]. Indeed, ATF4 has been shown to regulate starvation-induced skeletal muscle atrophy and promote the expression of autophagy genes [46]. Additionally, activation of the eIF2α/ATF4 signaling axis has been linked to DOX-induced cardiotoxicity, with Wang et al. revealing that knockdown of eIF2α in cardiomyocytes is sufficient to prevent cellular ROS production and cell death following DOX exposure [47]. Contrary to these findings, our results show that the UPR is not activated in the soleus muscle following DOX administration, and that inhibition of autophagy in DOX-treated animals also does not affect this stress signaling pathway. Therefore, DOX-induced autophagy in the soleus muscle occurs via a mechanism independent of the ER stress response. 

### 4.3. Inhibition of Autophagy Reduced ROS Production and Improved Mitochondrial Efficiency

DOX localization to the mitochondria results in the supraphysiological production of ROS, leading to muscle damage and weakness [10,48]. Inhibition of DOX-induced mitochondrial ROS production in skeletal muscle is sufficient to prevent muscle atrophy and contractile dysfunction [21,22], and our results show that selective inhibition of autophagy prevents the pathological increase in mitochondrial ROS production and reduction in mitochondrial function. In this regard, a similar regulatory cross-talk between oxidative stress and autophagy was recently revealed to play a central role in disuse-induced skeletal muscle wasting [7]. However, although the mechanism for the autophagy inhibition-induced preservation of muscle CSA and specific force production following disuse appears to be related to changes in the rate of peroxisome degradation and catalase expression [7], prevention of DOX-induced muscle weakness was found to be related to the effects of autophagy on mitochondrial volume, as indicated by citrate synthase expression. Although additional measurements are needed, maintenance of basal ROS emission and RCR in the dnATG5-DOX group suggests that select inhibition of pathologically elevated autophagy in the soleus muscle may sustain adequate clearance of dysfunctional mitochondrial while promoting increased mitochondrial volume.

### 4.4. Autophagy Inhibition Enhanced Soleus Muscle Antioxidant Capacity in DOX Treated Animals

Cellular ROS levels are tightly regulated to prevent the induction of oxidative damage to healthy tissue. To achieve redox balance, skeletal muscle has been shown to coordinate an adaptive response to prevent oxidative stress by regulating the levels of endogenous antioxidant enzymes [49]. Contrastingly, several reports suggest that DOX not only increases ROS production within the muscle, but also reduces the expression of antioxidant enzymes [10,21,50]. We found that the mRNA expression of both GPX1 and SOD2 were significantly reduced following DOX administration. Additionally, our novel findings demonstrate that in the absence of excessive autophagy, antioxidant enzyme expression is upregulated in the soleus. This corresponds to the observed changes in citrate synthase as GPX1, SOD1, SOD2, and catalase are all localized within different compartments of the mitochondria [51]. Moreover, overexpression of antioxidant enzymes in striated muscle has been shown to exhibit cytoprotective benefits following DOX administration [52,53,54,55]. Specifically, overexpression of SOD2 in the heart effectively prevented mitochondrial and cardiac dysfunction after DOX treatment [56,57]. Furthermore, overexpression of catalase was also shown to protect against cancer chemotherapy-induced skeletal muscle dysfunction in a tumor model [58], as well as suppressing DOX-induced cardiotoxicity in the heart [59]. Therefore, increased antioxidant capacity may be required for the autophagy inhibition-induced prevention of mitochondrial ROS and preservation of soleus muscle function.

### 4.5. Autophagy Inhibition Led to Improved Mitochondrial Biogenesis and ROS Detoxification

A relationship between autophagy and PGC-1α has been described during cellular stress, showing that the rate of mitochondria degradation is inversely related to the expression of PGC-1α [60]. Our findings agree with this, as inhibition of autophagy increased soleus muscle transcription of PGC-1α and several downstream transcriptional regulators of mitochondrial biogenesis and ROS detoxification. This is significant because disruption to mitochondrial biogenesis has been shown to be critically involved in the etiology of DOX-induced muscle toxicity [48], and it has been proposed that upregulation of PGC-1α may prevent DOX-induced atrophy through its effects on mitochondrial dynamics and/or by contributing to the inhibition of Forkhead box O3a (FoxO3a) signaling [61,62]. Our results support the hypothesis that PGC-1α stimulates mitochondrial biogenesis via the enhancement of NRF1/2 transcription, which corresponds with the observed increase in citrate synthase in the absence of DOX-induced autophagy.

In addition, our data suggest that coordination between SIRT1/3, PGC-1α, and the NRF2-ARE signaling axis may also exert protective effects against DOX toxicity through the coactivation of several transcription factors necessary for ROS detoxification and improvement of mitochondrial oxidative capacity. Target genes include the antioxidant enzymes GPX1, SOD1, SOD2, catalase, HO-1, and the NAD(P)H dehydrogenase NQO1 [63,64,65], which were all upregulated in response to autophagy inhibition and whose changes in expression are consistent with the changes in SIRT1, PGC-1α, and NRF2 expression. Indeed, this complex transcriptional response to inhibition of DOX-induced autophagy has the potential to maintain skeletal muscle cellular homeostasis following the adverse effects of DOX. Therefore, our data contend that in the absence of excessive autophagy, upregulation of transcriptional regulators of mitochondrial biogenesis and antioxidant capacity play a crucial role in regulating mitochondrial function and redox balance within the soleus muscle.

## 5. Conclusions

These results from this study provide novel evidence that accelerated autophagy is required for the development of DOX-induced skeletal muscle dysfunction. Further, our work reveals that the potential mechanisms responsible for autophagy inhibition-induced prevention of DOX myotoxicity are associated with upregulation of transcriptional regulators of mitochondrial biogenesis and the antioxidant stress response, which is sufficient to prevent mitochondrial ROS production and mitochondrial damage. Therefore, therapies targeting the pathological upregulation of autophagy following DOX treatment may effectively reduce the toxic effects of DOX on muscle tissue.

## Figures and Tables

**Table 1:** Differences in body weight, soleus muscle weight, and the ratio of soleus muscle weight to body weight between experimental groups in experiment 2.

	Saline-Saline	Saline-DOX	dnATG5-DOX
Initial weight (g)	278.6 ± 3.34	283.13 ± 5.52	280.13 ± 3.38
Treatment weight (g)	296.5 ± 4.40	302.8 ± 5.06	299.88 ± 4.50
Final weight (g)	295.9 ± 4.52	287.13 ± 6.57	290.5 ± 6.27
Soleus (mg)	123.5 ± 2.78	125.9 ± 2.76	122.0 ± 2.34
Soleus/body weight (mg/g)	0.42 ± 0.01	0.44 ± 0.01	0.42 ± 0.01

Values are presented as means ± SEM.
